# Dynamic regulation of adipose tissue metabolism in the domestic broiler chicken – an alternative model for studies of human obesity

**DOI:** 10.1186/1753-6561-6-S3-P67

**Published:** 2012-06-01

**Authors:** Bo Ji, Joelle Dupont, Jean Simon, Sue Lamont, Arnold Saxton, Brynn Voy

**Affiliations:** 1 Department of Animal Science, University of Tennessee, Knoxville, TN 37996, USA; 2 Unité de Physiologie de la Reproduction et des Comportements (UMR85), Institut National de la Recherche Agronomique (INRA), 37380, Nouzilly, France; 3 Unité de Recherches Avicoles (U83), Institut National de la Recherche Agronomique (INRA), 37380, Nouzilly, France; 4 Department of Animal Science, Iowa State University, Ames, IA 40011, USA

## Background

The domestic chicken is an attractive, but underutilized, animal model for studies of adipose tissue biology, metabolism and obesity: 1.) like humans, chickens rely on liver rather than adipose tissue for the majority of de novo lipogenesis; 2.) quantitative trait loci (QTLs) linked to fatness in chickens contain genes implicated in human susceptibility to obesity and diabetes; 3.) chickens are naturally hyperglycemic and insulin resistant; and 4.) a broad selection of genetic models exhibiting a range of fatness are available. To date, however, little is known about regulation of adipose metabolism in this model organism.

## Materials and methods

Affymetrix arrays were used to profile gene expression in abdominal adipose tissue from broiler chickens fed ad libitum or fasted for five hours and from three distinct genetic lines with low (Fayoumi and Leghorn) or high (broiler) levels of adiposity. QPCR was used to validate microarray results for select genes. Western blotting was used to assay levels of signaling proteins. Tissue levels of beta-hydroxybutyrate were measured as an index of fatty acid oxidation using a colorimetric assay. Multiple testing was controlled using q-value. Mixed linear model and multivariate clustering analysis were implemented in SAS. The Database for Annotation, Visualization and Integrated Discovery (DAVID) v6.7 (http://david.abcc.ncifcrf.gov/) was used for Gene Ontology (GO) and KEGG pathway enrichment analyses.

## Results

A total of 1780 genes were differentially expressed in fasted vs. ad libitum fed (p<0.05) tissue after correction for multiple testing. Gene Ontology and pathway analyses, combined with Western blot validation, indicated significant effects on a broad selection of pathways related to metabolism, stress signaling and adipogenesis. In particular, fasting upregulated rate-limiting genes in both the mitochondrial and peroxisomal pathways of beta-oxidation. Enhanced fatty acid oxidation in white adipose tissue was further suggested by a significant increase in tissue content of the ketone beta-hydroxybutyrate. Expression profiles suggested that, despite the relatively brief duration of feed withdrawal, fasting suppressed adipogenesis; expression of key genes in multiple steps of adipogenesis, including lineage commitment from mesenchymal stem cells, were significantly down-regulated in fasted vs. fed adipose tissue. Interestingly, fasting increased expression of several inflammatory adipokines and components of the toll-like receptor 4 signaling pathway. Microarray analysis of Fayoumi, Leghorn and broiler adipose tissue revealed that genetic leanness shared molecular signatures with the effects of fasting. In supervised clustering analysis, fasted broiler chickens clustered with lean Fayoumi and Leghorn lines rather than with the fed broiler group, suggesting that fasting manipulated expression profiles to resemble those of the lean phenotype.

## Conclusions

Collectively, these data suggest that leanness in chickens is associated with increased fat utilization which, given the similarities between avian and human adipose tissue with regard to lipid metabolism, may have relevance for humans. The paradoxical increase in some inflammatory markers with an acute fast suggests that the dynamic relationship between inflammation and adipose metabolism may differ from what is observed in obesity. These results highlight chicken as a useful model in which to study the interrelationships between food intake, adipose development, metabolism, and cell stress.

